# Perceived control over falling: a qualitative study of psychological aspects in older adults with fall concerns

**DOI:** 10.1186/s12877-026-07784-1

**Published:** 2026-06-05

**Authors:** Kanjanaporn Tantrongphak, Duangporn Suriyaamarit, Kanthee Anantapong

**Affiliations:** 1 Faculty of Allied Health Sciences, Chulalongkorn University, Bangkok, Thailand; 2 Department of Psychiatry, Faculty of Medicine, Prince of Songkla University, Hat Yai, Songkhla, 90110 Thailand

**Keywords:** Falls, Frailty, Ageing, Muscle strength, Qualitative research

## Abstract

**Background:**

Concern about falling (CaF) is common in later life and can both protect against and contribute to functional decline. However, how older adults with CaF construct perceived control over falling, and how this shapes fall-related awareness and behaviours, remains underexplored. This study aimed to explore how community-dwelling older adults with CaF interpret fall experiences and physical ageing, and how these interpretations shape perceived control over falling, activity engagement, and fall-prevention behaviours.

**Methods:**

We conducted a qualitative study in urban community settings in Bangkok, Thailand. Purposive sampling was used to recruit community-dwelling older adults with CaF who varied in perceived control over falling. Semi-structured individual interviews examined fall experiences, perceptions of CaF, ageing, and everyday activities. Interviews were audio-recorded, transcribed verbatim, and analysed using reflexive thematic analysis to develop themes capturing how perceived control over falling is constructed and continually negotiated.

**Results:**

Twenty older adults participated. Five interrelated themes described how perceived control over falling is shaped: (1) falling experiences being attributed to internal or external controls, (2) initiating fear and its transformation into adaptive awareness, (3) fall control transcending physical ageing and health through acceptance and adaptation, (4) the interplay of life history, peers, and social expectations in shaping resilience, and (5) ongoing negotiation of perceived control, activity engagement, and fall-prevention behaviours. Older adults with higher perceived control tended to transform initial fear into “authentic” awareness, integrate falls into a coherent ageing narrative, and use proportionate safety strategies while maintaining valued activities. Those with lower perceived control more often reported persistent worry, external or fatalistic attributions, and over- or under-cautious behaviours that restricted participation and eroded confidence.

**Conclusions:**

Perceived control over falling among older adults with CaF is a dynamic, multidimensional process that extends beyond physical risk factors. Fall-prevention interventions should therefore move beyond purely physical components to incorporate psychological, social, and life-course dimensions, supporting adaptive appraisals, resilience, independence, and sustained engagement in daily life.

**Supplementary Information:**

The online version contains supplementary material available at 10.1186/s12877-026-07784-1.

## Introduction

Falls are a major health problem among older adults worldwide and can negatively affect health, well-being, and quality of life [1–4]. In addition to physical injuries, falls can cause psychological problems, including in those without a history of falling [5–8]. A common psychological outcome is concern about falling (CaF) [9, 10], which often persists and is linked to activity avoidance, impaired balance, and increased risk of future falls [11–13]. However, CaF can also promote adaptive behaviours when individuals regulate their emotions and consistently use protective fall-prevention strategies [14–19]. Perceived control is a key psychological factor shaping emotional, physiological, and behavioural responses to the perceived threat of falling and is informed by prior health performance and outcomes. Individuals with higher perceived control tend to have better physical health, mental health, and overall well-being [20–22].

Perceived control over falling is a key determinant of whether CaF is adaptive or maladaptive [21]. It reflects one’s sense of control over threatening situations, including (i) the ability to manage balance and prevent falls and (ii) the capacity to channel fear into safer behaviours. Thus, individuals with high perceived control can modulate high CaF through conservative behavioural adaptations that enhance safety. In contrast, low perceived control can amplify emotional reactions, leading to an overestimation of harm, overly cautious movement, and impaired cognitive and attentional control of motor performance, resulting in inappropriate motor behaviours, heightened distractibility by external threats, and unnecessary activity restriction [23]. Alternatively, low perceived control may foster a sense of fatalism; when older adults perceive fall prevention as futile, they may disregard necessary safety precautions and persist in risky behaviours, further increasing their objective fall risk [21, 24]. These adverse consequences underscore how low perceived control shapes CaF and dictates whether behavioural responses become maladaptive [16].

Perceived control generally follows a non-linear lifespan trajectory, increasing through early adulthood, peaking in midlife, and declining in later life as control expectations and self-efficacy diminish alongside age-related biological, cognitive, and social change [20, 25, 26]. A substantial body of work shows that perceived control is shaped by demographic, socioeconomic, and cultural factors and is associated with physical performance, cognitive functioning, and psychological outcomes in later life [21, 22]. These findings highlight the importance of individual differences in perceived control among older adults with CaF and help explain why some experience serious consequences while others do not after similar fall events.

A recent meta-aggregation of qualitative studies on older adults with a history of falls described diverse perspectives and experiences, contrasting those who adopt a proactive, adaptive stance with those who enter a cycle of self-blame, activity curtailment, and dependence [27]. Similarly, qualitative evidence on worry about falling indicates that people interpret and respond to this fear differently, depending on their perceived ability to regulate emotional and physical responses to minimise risk [16]. However, mechanistic insight into the psychological processes underlying disparities in perceived control among those with fall concerns remains limited, even though perceived control is a highly individual, context-dependent construct that critically determines whether CaF operates as a protective or maladaptive mechanism. Consequently, this study used a qualitative approach to explore in depth how older adults’ experiences and perceptions shape their perceived control over falling among those with CaF.

## Materials and methods

### Design

A qualitative approach was employed, involving semi-structured individual interviews with older adults who had CaF. We analysed data using reflexive thematic analysis [28, 29] guided by a theoretical framework of the Perceived Control Model of Falling [23], which proposes perceived control as a central mediator of physiological, psychological, and behavioural responses to the perceived threat of falling, determining whether CaF functions adaptively or initiates a maladaptive cycle that increases fall risk. This study was reported in accordance with the Consolidated Criteria for Reporting Qualitative Research (COREQ) guidelines [30] (Appendix 1).

### Sampling and participant recruitment

This qualitative study was a pre-planned component of a larger mixed-methods project. Data were collected between June and November 2025 through individual semi-structured interviews with twenty purposively selected participants from a cross-sectional study of 550 community-dwelling older adults in Bangkok, Thailand, which examined sociodemographic, health, physical performance, and cognitive-psychological factors associated with perceived control over falling. A purposive sample was selected using predefined eligibility criteria (Table 1) to enable in-depth exploration of individual perceptions and lived experiences while ensuring maximum variation in age, education, and gender [31]. Potential participants were approached face-to-face during the parent study, screened for eligibility via self-questionnaires and interviews by the first author (KT), a physical therapist with extensive clinical experience, and subsequently invited to take part in individual interviews; no participants declined or were excluded after screening. The researcher explicitly informed participants that their responses were confidential and would not affect their clinical care or treatment outcomes. The target population was Thai older adults aged 60–80 years residing in the Bangkok metropolitan region.

**Table 1:** Eligibility criteria for participants

Inclusion Criteria	
1. Reported concern about falling (CaF), indicated by a FES-I Thai score between 17 and 64 [32, 33].	
2. Independent activity in daily life screened by the Barthel ADL ≥ 12 points [34].	
3. Able to communicate in the Thai language.	

Exclusion Criteria	
1. No history of progressive neurological conditions that lead to disability, i.e., Parkinson’s disease or advanced dementia.	
2. No history of severe psychiatric disorders, i.e., panic, and psychosis.	

The sample size was guided by the information power model and evidence that 9–17 interviews can be sufficient for relatively homogeneous samples with narrow aims [35–37]. We therefore aimed to recruit 20 participants, including 10 with high perceived control and 10 with low perceived control. The Thai version of the perceived control over falling scale (UP-COF Thai) was administered to assess the extent to which participants felt they could manage or prevent potential falls (Appendix 2) [38]. This instrument consists of 4 items; each rated on a 6-point Likert scale (0–5). Total scores range from 0 to 20, where higher scores represent a higher perceived control over falling. The scale has been validated for use in older adults and showed good psychometric properties [38]. A cut-off score of ≤ 13 was used to classify participants as having low perceived control over falling.

After a detailed explanation of the procedures, all participants provided written informed consent and were informed that the interviewer (KT) was a physical therapist and doctoral researcher from Chulalongkorn University. The specific research interests were disclosed to ensure participants understood the context and purpose of the inquiry before the interview commenced.

### Data collection

Before interviews, participants completed a demographic questionnaire to collect data on age, gender, education, and general health status was also recorded, including medication, comorbidity, and fall history. Interviews were conducted by a researcher (KT) with six years’ clinical experience as a physical therapist working with older adults and formal qualitative interviewing training, under regular supervision from a physiotherapist with extensive clinical and research experience in older adults (DS) and a geriatric psychiatrist specialising in qualitative research and mental illness in this population (KA).

Each face-to-face interview lasted 40–60 min and was conducted in a quiet, convenient room at their community setting with no third parties present. The interview guide comprised open-ended questions on (i) perceptions and experiences of falls and CaF, (ii) perceived control over falling, and (iii) individual differences in perceived control (Appendix 3). It was developed from relevant literature and the conceptual framework and refined through team discussions. Each participant completed a single interview, and audio was recorded using participant codes only, and recordings were securely destroyed after study completion and not made public.

### Data analysis

Interview data were transcribed verbatim and analysed using reflexive thematic analysis [28, 29], supported by QSR International NVivo (version 1.7.2). Transcripts were not returned to participants for comment or correction, nor was formal member-checking of preliminary themes conducted. The researcher repeatedly read transcripts for familiarisation, then generated initial codes during data collection to capture emerging patterns across interviews. Codes were subsequently collated into candidate themes, which were reviewed, refined, and clearly defined through iterative team discussions.

### Reflexivity

The research team used a reflexive approach to enhance rigour and credibility. The multidisciplinary team comprised a physiotherapist (KT, female), a senior physiotherapist (DS, female), and a geriatric psychiatrist (KA, male), providing complementary clinical and qualitative expertise. They acknowledged that their professional backgrounds could shape data collection and analysis. Potential bias and clinical projection were addressed using several reflexive strategies. The primary researcher (KT) kept a reflexive journal, documenting immediate post-interview reflections on emotions, assumptions, and the influence of her clinical background. These notes were discussed in regular debriefing meetings with the research team and revisited during analysis to make assumptions explicit, provide situational context, and support a more transparent interpretation of the transcripts.

## Results

### Participant characteristics

Twenty older adults aged 60–80 years participated, with a mean age of 69.8 years (SD = 6.2). Most were female (75%), with males comprising 25%. Participants were evenly divided into high and low perceived control (UP-COF) groups. Most participants were well-educated (secondary or higher), managed one to two chronic conditions, and took one to two daily medications. Significant distinctions emerged regarding fall-related variables; the high perceived control group consisted mainly of non-fallers with low levels of concern about falling. In contrast, the low perceived control group demonstrated higher vulnerability, with reporting recurrent falls and experiencing moderate-to-high concern about falling. Despite these differences, more than half of the participants across both groups perceived their overall health as “good” (Table 2).

**Table 2:** Participants’ characteristics categorised by level of perceived control over falling

Characteristic	High Perceived Control (*n* = 10)	Low Perceived Control (*n* = 10)	Total (*n* = 20)
Age (years), M (SD)	70.7 (7.5)	68.9 (4.7)	69.8 (6.2)
Range	60–80	61–76	60–80
Gender, n (%)			
Female	5 (50.0%)	10 (100.0%)	15 (75.0%)
Male	5 (50.0%)	0 (0.0%)	5 (25.0%)
Concern about falling (FES-I), M (SD), n (%)	21.70 (8.67)	27.50 (7.21)	24.60 (8.31)
Low	6 (60.0%)	0 (0.0%)	6 (30.0%)
Moderate	3 (30.0%)	6 (60.0%)	9 (45.0%)
High	1 (10.0%)	4 (40.0%)	5 (25.0%)
Education level, n (%)			
Illiterate	0 (0.0%)	1 (10.0%)	1 (5.0%)
Elementary	1 (10.0%)	2 (20.0%)	3 (15.0%)
Secondary	6 (60.0%)	3 (30.0%)	9 (45.0%)
Higher education	3 (30.0%)	4 (40.0%)	7 (35.0%)
Number of comorbidities, n (%)			
1–2	6 (60.0%)	7 (70.0%)	13 (65.0%)
≥3	2 (20.0%)	2 (20.0%)	4 (20.0%)
None	2 (20.0%)	1 (10.0%)	3 (15.0%)
Number of medications used, n (%)			
1–2	7 (70.0%)	7 (70.0%)	14 (70.0%)
≥3	3 (30.0%)	1 (10.0%)	4 (20.0%)
None	0 (0.0%)	2 (20.0%)	2 (10.0%)
Perceived health status, n (%)			
Very good	3 (30.0%)	0 (0.0%)	3 (15.0%)
Good	7 (70.0%)	7 (70.0%)	14 (70.0%)
Fair	0 (0.0%)	1 (10.0%)	1 (5.0%)
Poor	0 (0.0%)	2 (20.0%)	2 (10.0%)
Fall history in previous 12 months, n (%)			
No falls	8 (80.0%)	2 (20.0%)	10 (50.0%)
1–2 falls	1 (10.0%)	6 (60.0%)	7 (35.0%)
≥3 falls	1 (10.0%)	2 (20.0%)	3 (15.0%)

*FES-I* Falls Efficacy Scale-International, Concerns were classified as: Low (16–19), Moderate (20–27), and High (28–64) [39]. *UP-COF* Updated Perceived Control over Falling scale, Perceived control was classified as: Low (≤ 13) and High (> 13). *SD* Standard Deviation

### Key findings

Participants characterised falls as sudden, unforeseen incidents, with the primary fall often serving as a critical catalyst for acknowledging their transition into older age. While levels of CaF varied, they did not directly determine an individual’s management capability. Instead, perceived control emerged as the central factor shaping both their interpretation of the fall event and their subsequent response.

Five overarching themes were identified as key influences on this perceived control over falling. For a comprehensive overview, these themes, sub-themes, and key interpretive details are summarised in Table 3. Each theme is explored through illustrative quotes within the main text. In reporting embedded illustrative quotations, first-person pronouns (e.g. ‘I’, ‘me’) were occasionally replaced with third-person forms in square brackets (e.g. ‘[they]’, ‘[their]’) for consistency with the third-person narrative and purely for stylistic and readability purposes, while remaining self-referential accounts.

**Table 3:** Summary of themes, subthemes, and key interpretive comparisons

Theme	Subtheme	Key Interpretive Details
1. Falling experiences being attributed to internal or external controls	• Firsthand learning • Negative emotional response • External attributions	High perceived control participants used falls as learning tools for caution. Low perceived control participants exhibited self-blame, persistent worry, and attributed falls to external factors like fate or environment.
2. Initiating fear and its transformation into adaptive awareness	• Adaptive awareness • Maladaptive worry • Emotional regulation	Fear acted as a catalyst for “adaptive awareness” in high perceived control individuals, promoting safer behaviours. In low perceived control individuals, fear transformed into persistent worry and excessive, restrictive caution.
3. Fall control transcends physical ageing and health through acceptance and adaptation	• Mastery over physicality • Bodily weakness as constraint • Normalising decline	Participants with higher perceived control viewed health as manageable through self-care, preserving independence. Low perceived control participants perceived bodily decline and weakness as inevitable and insurmountable barriers to control.
4. The interplay of life history, peers, and social expectations in shaping resilience	• Life history & self-reliance • Social roles & responsibility • Peer influence	Participants with high perceived control exhibited resilience, self-reliance rooted in past life experiences, and their roles as parents or providers motivated them to maintain independence. Low perceived control individuals often internalise past falls as personal failures, leading to self-blame and persistent worry. Negative peer experiences and vicarious observations of decline reinforced their beliefs regarding ageing and heightened fall risk.
5. Ongoing negotiation of perceived control, activity engagement, and fall-prevention behaviours	• Strategic movement • Maintaining valued activity • Activity restriction	All participants used some strategies to prevent falls, but the type and balance of strategies differed. Higher perceived control individuals maintained valued activities through proportionate safety measures. Lower perceived control individuals engaged in “excessive caution” and non-essential activity restriction.

#### Falling experiences being attributed to internal or external controls

Despite similar exposure to falls, whether personal or vicarious, participants differed markedly in how they interpreted these events. Injury severity and hospitalisation did not determine perceived control. Instead, perceptions and emotional responses after the fall distinguished those with high from those with low perceived control.

All participants generally tried to identify the causes of their falls and expressed a desire to be more cautious, as some older adults noted, *“It’s probably because of the way [they] walk. [Their] walking style doesn’t lift [their] feet high enough… [They]’ve noticed this because it has happened many times*,* [and they] almost fell*,* but luckily*,* they managed to catch themselves. This experience has made [them] more cautious”* (P9, Female, 65 years old). Older adults with higher perceived control reported that their understanding of fall risk was shaped mainly by firsthand experiences. They rarely considered the possibility of falling before the first incident, as they had felt physically stable and had no prior events to prompt concern. The initial stumble became a turning point, prompting them to examine why it happened and seek further information. Through this process, they developed greater awareness of risky movements and clearer strategies to prevent future falls.

*“I think that if it hasn’t happened yet*,* then I probably wouldn’t think anything of it*,* because I had no experience to make me think about it. I thought it wouldn’t happen to me*,* because when I do things*,* I feel steady…When I fell*,* I could quickly take a step and stop myself. That worked to some extent. Actually*,* learning from my own experience…Once I had the experience*,* I understood that doing this would keep me from falling; doing that would make me fall…Then I started looking for information afterward and became more careful afterward”* (P6, Female, 74 years old)

Those with low perceived control often reacted to past falls with negative cognitions, such as self-blame or excessive worry, and were unable to identify specific contributing factors, instead viewing falls as unpredictable and beyond personal control. Some participants explained, *“The issue of balance that [they] mentioned makes [them] anxious. After falling*,* [they] don’t know what to do*,* and [they] get a bit stressed with themselves*,* wondering why [they] fall. How could [they] fall on such a flat surface? [They’re] afraid and don’t want to fall again*,* so [they] worry and try to be more careful.”* (P20, Female, 76 years old). Several participants attributed falls to external conditions such as environmental hazards, floor surfaces, fate, or luck, and regarded falling as an inevitable aspect of ageing rather than a reflection of their physical capacity or balance.

*“While I was walking*,* I just fell. It was like something pushed me*,* and my hands crossed*,* and then I lifted*,* and the ground was also wet. My body isn’t very strong. When I make a wish*,* I thank the sacred powers for protecting me so that nothing serious happens. I think I’m fairly strong*,* but when it comes to falling*,* if it’s going to happen*,* it will happen. You can’t really prevent it. Don’t be overly afraid or worried about it”* (P17, Female, 68 years old)

In addition to their own fall history, participants’ perceptions were influenced by the fall experiences of other older adults shared by friends, neighbours, and wider social networks. These vicarious accounts heightened fall awareness for all participants, but those with low perceived control were more likely to view them as evidence of potentially severe or fatal outcomes, which further intensified their fear and worry about falling.

*“Seeing news images makes me feel worried*,* for example*,* the video clips that they send*,* and relatives who send me clips to watch and tell me stories. It makes me concerned…This kind of worry mostly comes from other people’s severe incidents*,* from what I have seen*,* and I feel*,* ‘I really don’t want that to happen to me.’ The word ‘fear’ here means that I have seen many such images*,* so I have to be careful. I think that one day it will probably happen to me.”* (P19, Female, 69 years old)

#### Initiating fear and its transformation into adaptive awareness

Participants in both high and low perceived control groups reported a wide range of emotional responses after their falls. However, the extent to which these emotions fostered adaptive awareness differed markedly between the groups.

Nearly all participants described fears of recurrent falls, injury, and wider consequences such as death, prolonged inactivity, activity restriction, burdening others, and loss of autonomy. Some participants said that *“[They’re] afraid of falling because if there is any problem with [their] legs*,* it will make [them] unable to walk normally*,* or [they] might become disabled. [They] don’t want to become bedridden*,* [and] it can have effects such as making the people around them become burdened”* (P12, Female, 70 years old). For some, this awareness was adaptive, promoting greater caution and more effective fall-prevention strategies. For others, it became maladaptive, marked by heightened worry, excessive avoidance, and a diminished sense of control.

The primary difference between groups lay in how emotions were appraised and managed. When fear or worry arose in situations perceived as balance threats, older adults with high perceived control expressed greater confidence in their functional abilities: *“[They were] afraid*,* but [they] still live their life normally. It just makes [them] more cautious than before”* (P8, Female, 63 years old). Their emotional responses rarely escalated to the point of impairing actions or decisions. Instead, they acknowledged and regulated their emotions, using fear as a prompt for increased caution and maintaining a balance between fear of falling and confidence in their abilities.

*“My fear of falling is about level 7–8*,* but I can control myself*,* probably to the same degree*,* which cancels out the fear”* (P7, Female, 71 years old)

Individuals with low perceived control often reacted with vivid worst-case imaginings of severe injury or catastrophic outcomes that they *“have heard about falls*,* like some people sprained their legs*,* some ended up bedridden. This makes [them] even more worried than before…and [their] physical condition is worse than before…So*,* [they] keep imagining what would happen if [they] suddenly became dizzy. What would [they] do? [Their] imagination just keeps going”* (P18, Female, 75 years old). Although they wished to be cautious, their worry intensified and did not translate into effective coping strategies, instead leading to unnecessary, excessive caution. Some reported uncertainty about how they would manage a fall if it occurred and described emotional numbness or detachment, reflecting a limited sense of control over fall risk.

*“I am a bit careful*,* but sometimes I get careless too. If I have to walk on a wet floor*,* I don’t really think much about it. I think that if I’m going to fall*,* then it will just happen. I think like that. When I walk*,* I just know that I must not fall…must not fall.”* (P17, Female, 68 years old)

#### Fall control transcends physical aging and health—embracing acceptance and adaptation

Participants identified age-related physiological decline and chronic conditions, such as vertigo, visual impairment, and lower-limb weakness, as key contributors to impaired balance. Interpretations of these limitations differed by perceived control. Those with high perceived control adopted an attitude of “informed acceptance,” recognising functional change as part of ageing and proactively adapting their movement: *“[Their] age already limits them automatically…The quickness and agility are much slower than before. And with [their] mindset and feelings*,* as [they] get older*,* instead of becoming more active*,* [they] end up being less agile than [they] used to be…and when [they] used to run*,* now [they] can’t run anymore*,* so [they]walk or fast walk instead.”* (P3, Male, 60 years old). They retained confidence in their remaining abilities and viewed active health management as central to maintaining independence.

*“We have chronic illnesses or complications*,* like high blood pressure*,* which I have*,* I think that this part I try to control as well. I’ve been able to control it for about 20 years now. As for the physical ability*,* balance*,* and things like that*,* they help make me stronger”* (P3, Male, 60 years old)

Some participants in this group highlighted relatively strong lower-limb strength or overall fitness as resources that bolstered their confidence: *“exercising [their] legs regularly with exercise machines. [Their] leg muscles are okay. [They] feel much more at ease when [their] leg muscles are strong.”* (P10, Male, 78 years old). Even while acknowledging limitations, they emphasised remaining physical capacities and drew on these strengths to sustain a sense of capability.

*But now that my hip is fractured and I can’t walk*,* I feel troubled. I feel stressed. I try to adjust myself to get better at doing some exercise…I will try to do my best. I think and look at myself: if I don’t walk*,* it will be difficult…I must walk. Even if it hurts a bit*,* I still have to walk.”* (P5, Male, 73 years old)

In contrast, most older adults with low perceived control recognised multiple bodily impairments and viewed their physical weaknesses as constraints, feeling less strong than healthier peers even when their performance remained relatively good. They tended to regard their health as a major, uncertain limitation that restricted everyday activities.

*“As I get older*,* I know that I can fall easily. My bones are starting to weaken*,* so of course my worry has increased…my legs*,* some days*,* they feel kind of unsteady*,* like an old person…Because my body*,* I don’t really know what will occur. Seeing myself like this*,* I know that I am not the same as before*,* not like a young person anymore. Some people even older than me can still do things very well.”* (P18, Female, 75 years old)

Among older adults with low perceived control, two orientations emerged. One group overemphasised age-related bodily limitations, while the other normalised physical decline and fall risk as inevitable aspects of ageing. Although both acknowledged bodily change, the former fixated on their limitations, whereas the latter minimised these concerns and continued daily activities much as before.

*“The back pain and knee pain. I’m starting to get used to them now. Sometimes I just don’t pay attention to it anymore… It’s pretty much the same for all of us. It’s like*,* when you get older*,* you’re bound to have these things. It probably won’t get any better than this because I’m already quite old…”* (P11, Female, 70 years old)

#### Interplay of life history, peers, and social expectations in shaping resilience

Beyond personal factors, life histories and social contexts also shaped perceived control over falling. A prominent strength among older adults with high perceived control was resilience and adaptability. Many stated that they “*already have to rely on [themselves]. They shouldn’t think that they can depend on other people*,* [and they] must rely on themselves as much as [they] can.”* (P4, Male, 73 years old). They stressed persistence and self-reliance, aiming to stay active and maintain routines similar to earlier life. Many linked their resilience and confidence to earlier hardships that had forced them to become strong and adaptable, as one participant said, *“[He] was an orphan since [he] was young. [He was] under a lot of pressure…That’s why [he was] a strong person.”* (P4, Male, 73 years old). Social roles, such as being a parent or primary income earner, also reinforced the need to remain capable and avoid becoming a burden, as falls or functional decline could disrupt these responsibilities.

*“Families and children all have their own responsibilities. So*,* we have to try to take care of ourselves and help ourselves as much as possible…I try to tell myself that I don’t want to be a burden to my children and grandchildren. I want to be able to take care of myself*,* yes*,* as much as possible”* (P9, Female, 65 years old)

In contrast, older adults with low perceived control often showed limited adaptability and heightened anxiety. Some linked past mistakes to a long-standing tendency that undermined their confidence, ruminating over major work and lifestyle decisions. Others reflected on previous falls by recognising inattention and overestimation of their abilities, revealing a pattern of self-blame and persistent worry.

*“When I first fell*,* I concluded for myself that I had no awareness…I should have looked and seen that the steps weren’t the same height*,* so I should have taken a bigger step. But I had no awareness. Why do others run up and down just fine*,* while I stumble? I thought about it myself*,* so I said it was because I lacked awareness. I blamed myself like that.”* (P18, Female, 75 years old)

Social influences also shaped perceived control, as participants described adopting attitudes similar to those of their peers. For instance, *“[Those with high perceived control over falling] enjoy spending time with friends and regularly engage in shared activities such as working*,* traveling*,* exercising*,* and mountain climbing. [Their] friends always take [them] everywhere and encourage participation in these pursuits”* (P9, Female, 65 years old). In contrast, some with low perceived control frequently saw peers experience falls or chronic conditions such as knee pain or other non-communicable diseases, which they viewed as normal aspects of ageing. This sense of similarity with peers reinforced the belief that physical decline was inevitable and not amenable to change.

*“Most of us*,* being in our seventies*,* have some problems; people have knee issues…So*,* they have problems too. People our age mostly experience falls. They warn me to be careful*,* not to fall…People in the same age group are like me”* (P20, Female, 76 years old)

#### Ongoing negotiating perceived control, activity engagement, and fall-prevention behaviours

Perceived control over falling, activity engagement, and fall-prevention behaviours functioned as dynamic, interrelated processes. Low perceived control fostered maladaptive strategies and activity restriction, which in turn further reduced perceived control. Although all participants described using movement strategies to protect themselves from falls, maladaptive strategies were associated with increased inactivity among those with low perceived control.

Among older adults with low perceived control, even routine activities such as climbing stairs or walking on wet surfaces were perceived as highly hazardous. This led to excessive compensatory strategies, including constant use of handholds, frequent pauses, and reliance on physical support.

*“When going up a bridge*,* just a normal bridge. I feel that going up is manageable*,* but when coming down*,* will I slip down or not? I have already had this kind of fear since I was a teenager. Sometimes I must tell my friend*,* ‘Come help hold my hand*,*’ things like that*,* even though the surface is flat”* (P18, Female, 75 years old)

In unfamiliar environments or poor lighting, perceived control over falling was easily reduced, leading participants to avoid situations they viewed as risky and to forgo activities they considered non-essential, as one participant said, *“Sometimes [he] can’t see clearly*,* so if it’s not necessary*,* [he doesn’t] want to go anywhere”* (P14, Male, 71 years old). In contrast, those with high perceived control continued daily activities using proportionate bodily strategies and did not allow fear to dominate. Consistent physical activity and exercise were central for this group; they maintained usual activities, exercised regularly, and viewed these behaviours as essential for preserving health, believing that inactivity increased vulnerability to future falls and loss of independence.

*“Anything that can prevent falling*,* I try to do it every day…so I do it at home. At the Senior Club*,* they teach krabi-krabong (a traditional Thai weapon-based martial art developed for ancient battlefield combat)*,* and I also play the recorded clips I made*,* like aerobics*,* and I follow along. And I also do about 50 squats to make my legs strong. I think this is the most important. It’s better than doing nothing at all; otherwise*,* my confidence would be low. At least I can feel confident that my legs are much stronger because I have been doing many different things”* (P6, Female, 74 years old).

Older adults with low perceived control acknowledged the importance of physical activity, but the intensity and frequency of their exercise and daily activities were often too low to confer substantial benefit. Many avoided routine activities they previously managed because of fears of high-risk situations or recurring falls, as one participant stated *“Post-fall*,* [she] won’t enter wet or slippery fresh markets if it’s not essential*,* though [she] managed it previously”* (P11, Female, 70 years old). Some participants believed that *“Staying at home is good and safe”* (P11, Female, 70 years old). These patterns indicate that those with low perceived control did not consistently prioritise physical activity, reducing opportunities to improve physical capacity and lower fall risk.

## Discussion

This study deepens the understanding of how older adults with CaF construct perceived control over falling by examining the meanings they attach to fall-related experiences and concerns. A significant contribution of this work is offering a new perspective from a non-Western population, extending the qualitative evidence base that has been predominantly established within Western cultural contexts [16, 23]. Our findings enrich this existing framework by highlighting that fall interpretations vary widely and are shaped by an inclusive set of interacting factors spanning personal, physical, and socio-cultural dimensions. By moving beyond traditional CaF measures, this study provides a more comprehensive framework for understanding psychological resilience.

### Determinants of perceived control: integrating cognitive appraisals of falling with fall experiences, physical capacity, and psychological adaptability

Contrary to evidence suggesting that fall history inherently heightens concern or awareness [40–42], our findings suggest that it is not a definitive determinant of perceived control. Instead, cognitive and emotional appraisals of fall events— whether personally experienced or observed—primarily shape an individual’s perceived control and lead to either adaptive or maladaptive responses [16, 43–45]. Such appraisals help explain why older adults with similar levels of concern (CaF) and fall histories can differ markedly in their perceived control over falling [14–18]. Consistent with Rotter’s Locus of Control framework [46], participants with an internal locus of control employed constructive appraisals to identify risk factors and implement preventive strategies [27, 47]. By contrast, older adults with low perceived control often viewed falls as beyond personal control, focusing on external factors such as unfamiliar environments, chance, or fate. In the Thai context, Buddhist beliefs and related ideas such as karma may encourage attributing such events to higher powers or the consequences of past actions [48]. Consequently, some older adults regarded falls as an inevitable consequence of ageing, reducing motivation to explore causal factors or adopt adaptive safety behaviours [16, 49].

Similarly, physical capacity was often transcended by cognitive appraisal. Older adults can maintain a sense of mastery or self-efficacy [50], by adjusting the standards by which they judge their competence. Crucially, perceived control over falling is a domain-specific manifestation that interacts with broader trait characteristics. Consistent with Lachman’s (2011) framework [21], older adults may protect their global ‘trait’ control through goal-relevance shifting. When physical capacity declines, individuals often ‘downsize’ the importance of the fall-prevention domain. This strategic reappraisal prevents a situation-specific loss of control from damaging their overall, life-wide perception of mastery. Participants with high perceived control acknowledged age-related limitations but did not view them as insurmountable barriers, whereas those with low perceived control often showed a mismatch between perceived and actual performance, overestimating their weakness or exhibiting an underconfident appraisal of their capacity, a pattern linked to higher fall risk [18, 51]. Notably, these appraisals are not necessarily a mere byproduct of a fall experience; instead, they function within a bidirectional relationship where interpretations of falls (i.e., controllable vs. uncontrollable causes) establish perceived control levels, while baseline perceived control—as a resource—modulates emotional responses and coping strategies for future falls [20, 21].

Furthermore, our findings also suggest that perceived control is influenced by enduring traits such as resilience and adaptability [52–55], rooted in formative life experiences, social roles, community and social contexts, and independence-oriented lifestyles, which fostered adaptive coping and reinforced self-confidence in managing falls [21, 56]. In contrast, participants with low perceived control tended to be more pessimistic and anxious, marked by persistent worry and self-blame, consistent with evidence that lower control is associated with heightened stress responses [23, 25]. Interestingly, gender differences likely contribute to this pattern. Prior literature shows that older women report a higher sense of control than men [57, 58]. Male gender roles often encourage the underreporting of vulnerability, whereas older women may be more realistic or expressive about their physical limitations. This vulnerability pattern aligns with our findings, where low perceived control was concentrated solely among females.

### The role of perceived control in mediating authentic awareness and adaptive behaviours among older adults

Although fall-related awareness—typically expressed as increased caution and fear after a fall—is commonly reported among older adults [24, 59], our findings suggest that its usefulness depends on perceived control. Participants with high perceived control demonstrated “authentic awareness,” whereby fear became a catalyst for adaptive behaviour [16]. In contrast, those with low perceived control struggled to manage fear, allowing negative thoughts about potential outcomes to dominate, which disrupted conscious movement, diverted attention to negative thoughts or external threats, and limited effective engagement in protective behaviours [14, 23, 60, 61]. As a result, the “caution” they described often reflected superficial or unnecessary awareness rather than a meaningful guide to prevention, and in some cases was accompanied by limited or denied fear and minimal expressed concern about falling [24, 49].

Most older adults with low perceived control responded by avoiding situations they perceived as hazardous and withdrawing even from routine activities, consistent with the perceived control over falling framework [23]. This pattern contributed to physical inactivity, limited opportunities to build capacity, and reduced potential to prevent future falls. In contrast, those with high perceived control maintained activity and showed greater motivation, with ongoing engagement in more advanced physical activity creating a self-reinforcing cycle of health and functional performance. These findings are consistent with the multidirectional model of control beliefs [21], in which perceived control both arises from and shapes future performance through behavioural and emotional pathways.

### Clinical implication

Based on our findings, older adults with CaF interpret their fall experiences and emotional responses in diverse ways, resulting in varying levels of risk awareness and distinct behavioural patterns. These findings suggest that fall-prevention strategies may benefit from moving beyond traditional physical assessments (e.g., strength, balance, medication review, and environmental hazards) to further incorporate the psychological and contextual determinants of perceived control. This approach aligns with the international guidelines for falls prevention, which advocate for a multifactorial and person-centred perspective [1, 62].

Specifically, practitioners could utilise the UP-COF tool alongside standard CaF measures to help identify whether an older adult appraises their situation as controllable or uncontrollable. This assessment may aid in identifying those who require more than physical training, such as those exhibiting maladaptive worry. Accordingly, we propose a multidisciplinary approach where tailored exercise could be complemented by cognitive–behavioural elements. For instance, attribution retraining could help reframe unhelpful beliefs like fatalism or the overestimation of physical weakness. Furthermore, engaging family and community support ensures that these interventions are culturally sensitive and sustainable, particularly in contexts where social roles significantly shape an individual’s motivation for independence.

### Strength and limitation

This qualitative study provides an in-depth exploration of how fall experiences and CaF interpretations shape perceived control over falling across diverse contexts, offering a new perspective from a non-Western population. This contributes to a more nuanced understanding of perceived control that transcends Western-centric models. Methodological rigour was ensured through purposive sampling with maximum variation to enhance transferability, and researcher triangulation to strengthen credibility and dependability. Our findings reveal nuanced distinctions between older adults with high and low perceived control, highlighting how these perceptions regulate awareness of fall concerns and fall-prevention behaviours.

However, certain limitations should be acknowledged. The sample was drawn from an urban Thai setting, which may affect the transferability of the findings to rural populations or different cultural and religious contexts. While the Buddhist perspective on karma provided a unique lens for understanding the external locus of control, further research is needed to explore how other belief systems or cultural differences shape these psychological mechanisms. Additionally, beyond psychological dimensions, future studies should incorporate sociodemographic, physical, and cognitive variables for a more holistic view. Objective physiological data would further clarify whether participants possess a realistic perception of their control or exhibit an underconfident appraisal of their physical capabilities.

## Conclusion

Older adults with CaF interpret fall experiences and bodily ageing in diverse ways, shaping their perceived control over falling and, in turn, their fall-related awareness and behaviours. This study provides a theoretical basis for how perceptions and interpretations of fall experiences and CaF, appraisals of body and health, personal dispositions, and social influences together shape perceived control, highlighting the significant role of psychological factors in fall awareness and behaviour. Consequently, fall-prevention interventions for older adults with CaF should extend beyond physical components to more explicitly address older adults’ perceptions and lived experiences. Attending to these multiple dimensions offers a potential pathway to help maintain independence, enhance confidence, and support continued active engagement in daily life.

## Supplementary Information

Supplementary Material 1.

Supplementary Material 2.

Supplementary Material 3.

## Data Availability

The data that support the findings of this study are available from the corresponding author, upon reasonable request.
