# Supplementary material for: Perceived control over falling: a qualitative study of psychological aspects in older adults with fall concerns
Source: BMC Geriatr. 2026 Jun 5;26:1030. doi: 10.1186/s12877-026-07784-1 (PMC13459381; doi:10.1186/s12877-026-07784-1)
Supplement: Supplementary file 1 [file 12877_2026_7784_MOESM1_ESM.docx]

**Consolidated Criteria for Reporting Qualitative Research (COREQ)**

*32-Item Checklist for Interviews and Focus Groups (Tong, Sainsbury & Craig, 2007)*

*Manuscript: Perceived Control over Falling: A Qualitative Study of Psychological Aspects in Older Adults with Fall Concerns*

Authors: Tantrongphak K, Suriyaamarit D, Anantapong K

| **No.** | **Item** | **Guide question/description** | **Page** | **Location and notes** |
| --- | --- | --- | --- | --- |
| **DOMAIN 1: RESEARCH TEAM AND REFLEXIVITY** | | | | |
| ***Personal Characteristics*** | | | | |
| 1 | Interviewer/facilitator | *Which author/s conducted the interview or focus group?* | pp. 6, 8 | KT (first author, physical therapist) identified as the interviewer at both eligibility screening and data collection |
| 2 | Credentials | *What were the researcher's credentials? E.g. PhD, MD* | pp. 1, 8 | All three authors hold PhD (title page, line 5). KT described as physical therapist with six years clinical experience and formal qualitative interviewing training |
| 3 | Occupation | *What was their occupation at the time of the study?* | pp. 1, 8–9 | KT = physical therapist, Faculty of Allied Health Sciences; DS = senior physiotherapist; KA = geriatric psychiatrist, Department of Psychiatry |
| 4 | Gender | *Was the researcher male or female?* | p. 9 | Gender of all three researchers now stated in Reflexivity: KT (female), DS (female), KA (male) |
| 5 | Experience and training | *What experience or training did the researcher have?* | p. 8 | KT: six years clinical experience with older adults, formal qualitative interviewing training, under supervision of DS and KA |
| ***Relationship with Participants*** | | | | |
| 6 | Relationship established | *Was a relationship established prior to study commencement?* | p. 8 | Participants were from a larger cross-sectional study; KT conducted eligibility screening. Confidentiality of responses was explicitly disclosed to participants prior to commencement |
| 7 | Participant knowledge of the interviewer | *What did the participants know about the researcher? E.g. personal goals, reasons for doing the research* | p. 8 | Participants were informed that KT was a physical therapist and doctoral researcher from Chulalongkorn University, and the specific research interests were disclosed before the interview commenced. |
| 8 | Interviewer characteristics | *What characteristics were reported about the interviewer/facilitator? E.g. Bias, assumptions, reasons and interests in the research topic* | p. 9 | Reflexivity section describes professional backgrounds and potential bias; regular debriefing and reflexive journaling used as mitigation; KT documented reflections immediately after each interview. |
| **DOMAIN 2: STUDY DESIGN** | | | | |
| ***Theoretical Framework*** | | | | |
| 9 | Methodological orientation and Theory | *What methodological orientation was stated to underpin the study? E.g. grounded theory, discourse analysis, ethnography, phenomenology, content analysis* | p. 6 | Reflexive thematic analysis (Braun & Clarke [28, 29]) guided by the Perceived Control Model of Falling [23] explicitly stated as analytical and theoretical framework. |
| ***Participant Selection*** | | | | |
| 10 | Sampling | *How were participants selected? E.g. purposive, convenience, consecutive, snowball* | p. 6 | Purposive sampling with maximum variation (age, education, gender) clearly described; information power model cited for sample size justification. |
| 11 | Method of approach | *How were participants approached? E.g. face-to-face, telephone, mail, email* | p. 8 | Participants were contacted by face-to-face approach during the parent study, underwent eligibility screening, and were subsequently invited to participate by KT. |
| 12 | Sample size | *How many participants were in the study?* | pp. 6, 9 | Target of 20 participants (10 high, 10 low perceived control) stated in Sampling section; confirmed in Results. |
| 13 | Non-participation | *How many people refused to participate or dropped out? Reasons?* | p. 8 | Now explicitly stated: no participants declined the invitation or were excluded after the screening process. |
| ***Setting*** | | | | |
| 14 | Setting of data collection | *Where was the data collected? E.g. home, clinic, workplace* | p. 8 | Each interview was conducted in a quiet, convenient room at their community setting. |
| 15 | Presence of non-participants | *Was anyone else present besides the participants and researchers?* | p. 8 | Now explicitly stated: interviews were conducted with no third parties present. |
| 16 | Description of sample | *What are the important characteristics of the sample? E.g. demographic data, date* | pp. 9–11  Table 2 | Table 2 provides full demographic characteristics: age, gender, education, comorbidities, medications, perceived health, fall history, and CaF level by group. |
| ***Data Collection*** | | | | |
| 17 | Interview guide | *Were questions, prompts, guides provided by the authors? Was it pilot tested?* | p. 8 | Interview guide with open-ended questions on three domains described (Appendix 2); developed from literature and conceptual framework, refined through team discussions. Pilot testing not explicitly stated. |
| 18 | Repeat interviews | *Were repeat interviews carried out? If yes, how many?* | p. 8 | Now explicitly stated: each participant completed a single interview. |
| 19 | Audio/visual recording | *Did the research use audio or visual recording to collect the data?* | p. 8 | Audio recorded using participant codes only; recordings securely destroyed after study completion. |
| 20 | Field notes | *Were field notes made during and/or after the interview or focus group?* | p. 9 | Reflexive journaling conducted by KT immediately after each interview; notes used to manage researcher bias and provide situational context during interpretation. |
| 21 | Duration | *What was the duration of the interviews or focus group?* | p. 8 | Each interview lasted 40–60 minutes. |
| 22 | Data saturation | *Was data saturation discussed?* | p. 6 | Sample size guided by information power model; prior work suggesting 9–17 interviews for homogeneous samples cited as saturation rationale. |
| 23 | Transcripts returned | *Were transcripts returned to participants for comment and/or correction?* | p. 9 | Now explicitly stated: transcripts were not returned to participants for comment or correction, and formal member-checking of preliminary themes was not conducted. |
| **DOMAIN 3: ANALYSIS AND FINDINGS** | | | | |
| ***Data Analysis*** | | | | |
| 24 | Number of data coders | *How many data coders coded the data?* | p. 9 | Initial coding conducted by KT; candidate themes reviewed and refined through iterative team discussions with DS and KA. Independent coding by multiple analysts not explicitly stated. |
| 25 | Description of the coding tree | *Did authors provide a description of the coding tree?* | pp. 9, 12–13  Table 3 | Five themes and subthemes presented in Table 3; analytical process described. |
| 26 | Derivation of themes | *Were themes identified in advance or derived from the data?* | pp. 6, 9 | Themes derived inductively from data using reflexive thematic analysis within the theoretical framework of the Perceived Control Model of Falling. |
| 27 | Software | *What software, if applicable, was used to manage the data?* | p. 9 | QSR International NVivo (version 1.7.2) explicitly named. |
| 28 | Participant checking | *Did participants provide feedback on the findings?* | p. 9 | Now explicitly stated: formal member-checking of preliminary themes was not conducted. Rigour maintained through researcher triangulation and reflexive journaling. |
| ***Reporting*** | | | | |
| 29 | Quotations presented | *Were participant quotations presented to illustrate the themes/findings? Was each quotation identified? E.g. participant number* | pp. 14–22 | Rich illustrative quotations provided for all five themes; each attributed with participant code, gender, and age (e.g. P6, Female, 74 years old). |
| 30 | Data and findings consistent | *Was there consistency between the data presented and the findings?* | pp. 14–26 | Quotations are closely aligned with theme descriptors in Table 3 and interpretive discussion. |
| 31 | Clarity of major themes | *Were major themes clearly presented in the findings?* | pp. 11–22 | Five major themes clearly named, summarised in Table 3, each elaborated with subthemes and illustrative quotations in Results, and integrated into Discussion. |
| 32 | Clarity of minor themes | *Is there a description of diverse cases or discussion of minor themes?* | NA | No cases were found to fundamentally contradict the central themes; however, all minor variations and divergent orientations within themes were documented and analyzed to ensure the depth and breadth of the findings. |

**Reference:** Tong A, Sainsbury P, Craig J. Consolidated criteria for reporting qualitative research (COREQ): a 32-item checklist for interviews and focus groups. *Int J Qual Health Care*. 2007;19(6):349–357. |
