# Supplementary material for: Perceived control over falling: a qualitative study of psychological aspects in older adults with fall concerns
Source: BMC Geriatr. 2026 Jun 5;26:1030. doi: 10.1186/s12877-026-07784-1 (PMC13459381; doi:10.1186/s12877-026-07784-1)
Supplement: Supplementary file 2 [file 12877_2026_7784_MOESM2_ESM.docx]

**Appendix 2**

**The Thai version of UP-COF**

**Reference:** Tantrongphak, K., Anantapong, K., & Suriyaamarit, D. (2025). The Thai Version of the Updated Perceived Control Over Falling Scale: A Study of Cross-Cultural Adaptation, Reliability, and Validity in Older Adults. Journal of Health Science and Medical Research, 0, e20251268. doi:http://dx.doi.org/10.31584/jhsmr.20251268
