# Supplementary material for: Perceived control over falling: a qualitative study of psychological aspects in older adults with fall concerns
Source: BMC Geriatr. 2026 Jun 5;26:1030. doi: 10.1186/s12877-026-07784-1 (PMC13459381; doi:10.1186/s12877-026-07784-1)
Supplement: Supplementary file 3 [file 12877_2026_7784_MOESM3_ESM.docx]

**Appendix 3**

**The topic questions guide in an interview guide**

**Individual’s perception and experiences of fall and the emergence of CaF**

(1) Can you share any experiences or incidents related to falling, near falls, or situations where you felt unsteady? (If not, ask about the experiences of people around them who have fallen.)

(2) Do these incidents make you concern about falling? How so? (Rate the level of concern on a scale of 1 to 10.)

What are you worried or concerned about?

When did these worries start? What triggered your concerns?

(3) How do feelings affect your daily life? Does it make you avoid certain activities? How so?

If mentioning avoidance: What activities do you avoid? How does avoiding these activities make you feel? What do you think would happen if you participated in these activities?

What do you feel exacerbates your feelings?

**Perceived control over their falling situation**

(1) Do you feel confident when you're in situations where you might fall or feel unsteady? Do you feel in control? How so? (Try to imagine a scenario where you are about to fall or stumble.)

(2) How does this anxiety impact your daily life? For example, does it make you feel stressed, anxious, or discouraged (emotionally/mentally)?

(3) Do you think this concern affects your balance or walking?

(4) When you feel concerned, what do you do to try to overcome or reduce feelings? (For example, trying not to be afraid, feeling body stiff, looking for light, watching the ground, finding a safe path, looking for something to hold onto, etc.)

(5) Can you describe the ways you use to reduce your concern? How do you feel about your management strategies?

**Individual differences in the perspective of perceived control**

(1) **Relevant goals**
    - Do you think controlling yourself to prevent falls is important? How so?
(2) **Mix of gain and loss of control belief domains**
    - What obstacles do you face in terms of movement or balance, or what increases your concern about falling? (For example, decreased balance, weakened muscles, poor eyesight, etc.)
    - How have you adapted to these concerns?
(3) **Perceived control variability**
    - Compared to before, has your anxiety increased? How so?
    - Do you feel you can control yourself to avoid falling as much as you used to? How so?

**Additional Comments:**
Do you have any additional comments or anything you would like to add about your experience?
